# Comparison between Aptima Assays (Hologic) and the Allplex STI Essential Assay (Seegene) for the diagnosis of Sexually transmitted infections

**DOI:** 10.1371/journal.pone.0222439

**Published:** 2019-09-12

**Authors:** Adolfo de Salazar, Beatriz Espadafor, Ana Fuentes-López, Antonio Barrientos-Durán, Luis Salvador, Marta Álvarez, Federico García

**Affiliations:** 1 Hospital Universitario San Cecilio, Servicio de Microbiología, Instituto de Investigación Ibs, Granada, Spain; 2 Hospital Universitario Virgen de las Nieves, Servicio de Dermatología, Centro de ETS, Granada, Spain; GGD Amsterdam, NETHERLANDS

## Abstract

Sexually transmitted infections (STIs) remain a worldwide problem and a severe threat to public health. The purpose of this study was to compare Aptima^®^ Assays (Hologic^®^) and the Allplex^™^ STI Essential Assay (Seegene^®^) for the simultaneous detection of *Chlamydia trachomatis*, *Neisseria gonorrhoeae*, *Trichomonas vaginalis* and *Mycoplasma genitalium* in clinical practice. The Aptima^®^ assays (Hologic^®^) are based on a transcription-mediated amplification (TMA) method. The Allplex^™^ STI Essential assay (Seegene^®^) is based on a multiplex Real-Time PCR (RT-PCR) method. A total of 622 clinical samples from different anatomical sites were tested using both methods. A total of 88 (14.1%) and 66 (10.6%) positive samples were found for any of the TMA assays used and for the RT-PCR assay, respectively. Aptima^®^ assays showed a slightly higher rate of positive results for all pathogens except for *T*. *vaginalis*, the results of which were similar to those obtained with Allplex^™^. The most commonly detected pathogen was *C*. *trachomatis* (37 samples; 5.9% using TMA assays) and the anatomical site with the highest prevalence of microorganisms was a non-urogenital site, the pharynx (27 positive samples; 4.3%). Using the Aptima^®^ assays as reference method, the comparison showed that the average specificity of multiplex RT-PCR was 100.0% for the four pathogens. However an average sensitivity of 74.5% was observed, showing 95.2% (CI95%; 93.6–96.9) of overall concordance (*κ* = 0.80). In conclusion, the Aptima^®^ assays show a higher sensitivity on a wide range of sample types compared to the Allplex^™^ assay.

## Introduction

Sexually transmitted infections (STIs) remain a worldwide problem and a severe threat to public health. In 2012, approximately 130.9, 78.3, and 142.6 million new cases of *Chlamydia trachomatis*, *Neisseria gonorrhoeae*, and *Trichomonas vaginalis* infections, respectively, were estimated globally [1]. For *Mycoplasma genitalium*, prevalence has been estimated at 1.3% in high-income countries and 3.9% in low-income countries [2]. These microorganisms are responsible for a variety of clinical syndromes in women and men, such as urethritis, cervicitis, prostatitis and vaginitis [3–6], which may lead to severe complications and long-term sequelae, including pelvic inflammatory disease, infertility, chronic pelvic pain, ectopic pregnancy, neurological and cardiovascular disease in adults, premature delivery, neonatal death, severe disability or blindness in infants, and increased risk of HIV acquisition and transmission [7, 8].

Prompt recognition and appropriate treatment are essential for the control of transmission of STIs, and this requires sensitive and accurate laboratory diagnostic methods. The implementation of nucleic acid amplification tests (NAATs) has revolutionized diagnostics in the detection of *C*. *trachomatis*, *N*. *gonorrhoeae*, *T*. *vaginalis* and *M*. *genitalium*, due to their numerous advantages over conventional methods [9]. However, these tests traditionally required separate processes for nucleic acid extraction and amplification/detection, which may increase the risk of errors (manipulation errors, contamination during the extraction step, etc.), especially in high-volume diagnostic laboratories. Another limitation is the need for batch samples, which may delay diagnosis and treatment initiation.

The use of multiplex RT-PCR for the diagnosis of STI is widespread in Microbiology laboratories, because they offer a large panel of microorganisms in a simple reaction, at a low cost. However, these techniques may have a lower sensitivity than others commercially available.

Currently there are assays for the diagnosis of STI based on transcription-mediated amplification (TMA) targeting directly ribosomal RNA (rRNA), with the advantage of a higher number of copies per cell compared to DNA-based tests, which only target one copy. Assays based on TMA potentially increase sensitivity of detection compared to assays based on RT-PCR targeting single-copy genes [10–15]. To date, only a few studies compare these two methodologies for the simultaneous diagnosis of the 4 most important pathogens causing STIs [16, 17].

The objective of this study was to assess the performance of multiplex RT-PCR [Allplex^™^ STI Essential (Seegene^®^, Seoul, South Korea)]. This is an *in vitro* diagnostic (IVD) and CE-marked system for the simultaneous detection of *C*. *trachomatis*, *N*. *gonorrhoeae*, *T*. *vaginalis and M*. *genitalium*. The following FDA-cleared assays based on TMA were used as the reference method: Aptima Combo 2^®^ (for *C*. *trachomatis* and *N*. *gonorrhoea*), Aptima^®^ *T*. *vaginalis* and Aptima^®^ *M*. *genitalium* (Hologic^®^, San Diego, USA)].

## Material and methods

### Patients

The study was conducted between May 2017 and November 2017 in Granada, Spain. A total of 375 patients from the Centre for Sexually Transmitted Diseases in Granada were enrolled in the study. The median age of males (*n* = 243; 65%) and females (*n* = 132; 35%) was 29 years [IQR: 23–37]. The study was designed and conducted according to the principles expressed in the Declaration of Helsinki and it was approved by the local Ethics Committee of Hospital Universitario San Cecilio. Verbal informed consent was obtained from all participants.

Data underlying the findings described in this study have been deposited to Figshare and they are accessible via https://doi.org/10.6084/m9.figshare.9159746.v2. All other relevant data are shown in the present manuscript.

### Specimen collection

A total of 622 prospective clinical specimens from different anatomical sites (urine and endocervical, pharyngeal and anal swabs) according to the reported type of sexual practices (vaginal, oral and/or anal intercourse) of 375 participants were collected in duplicate. Table 1 shows a detailed description of the anatomical location of the collected samples. Specimens for routine testing were collected with dry swabs. The swabs were suspended in 2 mL of 1X Phosphate Buffered Saline solution (PBS) as transport system. Urine samples for TMA-assay testing were collected using the Aptima^®^ Urine Collection Kit for Male and Female Specimens (Hologic, San Diego, USA). Female endocervical and male urethral samples were collected with the Aptima^®^ Unisex Swab Specimen collection kit (Hologic, San Diego, USA). The Aptima^®^ Multitest Swab Specimen Collection Kit (Hologic, San Diego, USA) was used for the collection of pharyngeal and anal specimens. Random sampling was performed by alternating the collection of specimens for routine testing and specimens for Aptima^®^ testing. The distribution of the types of clinical specimens (622) was the following: 218 (35%) pharyngeal swabs, 214 (35%) first-void urine samples, 107 (17%) endocervical swabs and 83 (13%) rectal swabs. After collection, specimens were stored at 4°C until testing, generally, for two or three days after specimen collection. All NAATs were performed in parallel by the same technician.

**Table 1:** Distribution of the collected samples.

Anatomical site	Male n (%) of patients	Female n (%) of patients
Pharyngeal	16 (6.6%)	23 (17.4%)
Endocervical	-	22 (16.7%)
Urine	130 (53.5%)	0
Pharyngeal and Urine	37 (15.2%)	0
Anal and Urine	1 (0.4%)	0
Anal and Pharyngeal	14 (5.8%)	2 (1.5%)
Anal and Endocervical	-	3 (2.3%)
Endocervical and Urine	-	1 (0.8%)
Endocervical and Pharyngeal	-	63 (47.7%)
Endocervical and Pharyngeal and Urine	-	1 (0.8%)
Anal and Endocervical and Pharyngeal	-	17 (12.9%)
Anal and Urine and Pharyngeal	45 (18.5%)	0
**Total number of Patients (375)**	**243 (64.8%)**	**132 (35.2%)**
**Total number of Samples (622)**	**385 (61.9%)**	**237 (38.1%)**

### Real-Time multiplex PCR assay

Testing was performed using the multiplex RT-PCR Allplex^™^ STI Essential Assay (Seegene, Seoul, Korea). This assay can simultaneously detect 7 STI pathogens (*C*. *trachomatis*, *N*. *gonorrhoeae*, *T*. *vaginalis*, *M*. *genitalium*, *M*. *hominis*, *U*. *urealyticum* and *U*. *parvum*) in a single tube by using dual priming oligonucleotide (DPO^™^) and multiple detection temperatures (MuDT^™^) technologies, providing individual C_t_ values for multiple pathogens in a single channel. The DPO system differs structurally and functionally from the conventional primer system by including a poly deoxyinosine (I) linker, between two segments of primer sequences. This poly (I) linker allows dividing the DPO primer into two perfectly functional segments with different hybridization temperatures [18]. The elongation will be conducted when the two segments hybridize correctly giving rise to a high specificity between similar or related sequences. Previous nucleic acid extraction was performed (400 μL sample volume) using MagNA Pure 96 System (Roche); nucleic acids were eluted in 50 μL (final volume). Real-time PCR was performed in a CFX-96 real-time thermocycler (Bio-Rad, CA, USA), according to the manufacturer’s instructions. To maximize cost-efficiency without a significant delay in reporting, routine testing was performed in batches every Tuesday and Friday. For this study, only the results for *C*. *trachomatis*, *N*. *gonorrhoeae*, *T*. *vaginalis* and *M*. *genitalium* were analyzed.

### Transcription-mediated amplification assay

The Aptima^®^ assays comprise three main steps: target capture, TMA of the species-specific targets in the rRNA, and target detection by hybridization with complementary probes linked to chemiluminescent labels. The TMA step consists of a target nucleic acid amplification method using RNA transcription (RNA polymerase) and DNA synthesis (reverse transcriptase) to produce a RNA amplicon from a target nucleic acid; TMA can be used to target both RNA and DNA [19].

Aptima^®^ *M*. *genitalium* (MG), Aptima^®^ Combo 2 (detecting both *C*. *trachomatis*, *N*. *gonorrhoeae* in one sample) and Aptima^®^ *T*. *vaginalis* assays were used on the Panther^®^ system (Hologic, San Diego). This system is a fully automated testing platform with true sample-to-result automation allowing sample testing with Aptima^®^ assays, therefore avoiding the separate DNA extraction step. For this evaluation, samples collected for the Aptima^®^ assays were stored at 4°C and run on the same day as the RT-PCR.

### Data analysis

Sensitivity (SE), specificity (SP) and *kappa* coefficient (*κ*) of the multiplex RT-PCR were calculated and compared with TMA assays for detection of *C*. *trachomatis*, *N*. *gonorrhoeae*, *M*. *genitalium* and *T*. *vaginalis* using the statistical package SPSS, version 23 (IBM, Chicago, IL, USA). The corresponding two-tailed 95% score (Wilson) confidence intervals (CIs) were also estimated.

## Results

A total of 622 samples were tested using both methods. Positive results were found in 88 (14.1%) out of 622 samples for all the Aptima^®^ assays used. Regarding the Allplex^™^ assay, only 66 (10.6%) out of 622 samples showed positive results. Table 2 and S1 Table show the diagnostic performance of the systems used for detection of the different pathogens. The TMA-based assays performed on the Panther^®^ platform showed slightly higher positive results for *C*. *trachomatis*, *N*. *gonorrhoeae* and *M*. *genitalium*, while results for the *T*. *vaginalis* samples were similar for both RT-PCR (Allplex^™^) and TMA (Aptima^®^) assays. The most frequently detected pathogen was *C*. *trachomatis* (37 samples; 5.9%).

**Table 2:** Diagnostic performance of Allplex^™^ STI Essential assays in relation to the Aptima^®^ assays for *C*. *trachomatis*, *N*. *gonorrhoeae*, *M*. *genitalium* and *T*. *vaginalis*.

	Aptima^®^	Allplex^™^	DIAGNOSTIC PERFORMANCE		
PATHOGENS	Positive n (%)	Positive n (%)	SENSITIVITY % SE (C.I. 95%)	SPECIFICITY % SP (C.I. 95%)	Kappa (k)
*C*. *trachomatis*	37 (5.9%)	31 (5.0%)	83.8% (67.3–93.2)	100.0% (99.2–100)	0.91
*N*. *gonorrhoeae*	29 (4.7%)	21 (3.4%)	72.4% (52.5–86.6)	100.0% (99.2–100)	0.83
*M*. *genitalium*	24 (3.9%)	10 (1.6%)	41.7% (22.8–63.1)	100.0% (99.2–100)	0.58
*T*. *vaginalis*	5 (0.8%)	5 (0.8%)	100.0% (46.3–100)	100.0% (99.2–100)	1

**CI**, confidence interval.

The TMA-positive samples were further analyzed according to their anatomical site. Results revealed that a non-urogenital sample, the pharynx, was the most frequent site with a positive result (29 positive samples; 4.7%) followed by urine (27 positive samples; 4.3%), endocervical (22 positive samples; 3.5%) and anal samples (17 positive samples; 2.7%) (Tables 3 and 4). A total of 14 more samples were found positive for *M*. *genitalium* using Aptima^®^ assays (*n* = 24) than positive samples for *M*. *genitalium* identified using the Allplex^™^ STI Essential assay (*n* = 10); ten of these Aptima-positive samples were collected from pharyngeal swabs.

**Table 3:** Anatomical distribution and prevalence of the Aptima^®^-positive samples.

Site	*n*	*C*. *trachomatis* n (%)	*N*. *gonorrhoeae* n (%)	*M*. *genitalium* n (%)	*T*. *vaginalis* n (%)
**Anal**	83	6 (7.2%)	6 (7.2%)	4 (4.8%)	1 (1.2%)
**Endocervical**	107	12 (11.2%)	2 (1.9%)	4 (3.7%)	4 (3.7%)
**Pharyngeal**	218	9 (4.1%)	10 (4.6%)	10 (4.6%)	0.0%
**Urine**	214	10 (4.7%)	11 (5.1%)	6 (2.8%)	0.0%

**Table 4:** Anatomical distribution and prevalence of the Allplex^™^-positive samples.

Site	*n*	*C*. *trachomatis* n (%)	*N*. *gonorrhoeae* n (%)	*M*. *genitalium* n (%)	*T*. *vaginalis* n (%)
**Anal**	83	5 (6.0%)	6 (7.2%)	1 (1.2%)	1 (1.2%)
**Endocervical**	107	11 (10.3%)	1 (0.9%)	3 (2.8%)	4 (3.7%)
**Pharyngeal**	218	6 (2.8%)	8 (3.7%)	2 (0.9%)	0.0%
**Urine**	214	9 (4.2%)	6 (2.8%)	4 (1.9%)	0.0%

The diagnostic performance of the Allplex assay was evaluated by the calculation of SE and SP parameters. Concordance between the Aptima^®^ and the Allplex assays was determined through the calculation of the Cohen’s Kappa index, *κ* coefficient of the Allplex assay in relation to the Aptima^®^ assays (reference method) for the detection of *C*. *trachomatis*, *N*. *gonorrhoeae*, *M*. *genitalium* and *T*. *vaginalis* (Table 2). A specificity of 100.0% was found for the four pathogens, however an average sensitivity of 74.5% was observed for the Allplex assay. Both methods (TMA and multiplex RT-PCR) have shown a variable consistency depending on the microorganism detected, with κ values ranging between 0.58 for *M*. *genitalium* and 0.91 for *C*. *trachomatis*. In general, a concordance of 95.2% (CI95%; 93.6–96.9) and κ = 0.80 were obtained between both methods. Among the 622 samples, there were 28 discrepancies: 4 anal, 3 endocervical, 13 pharyngeal and 8 urine samples. Co-infections were observed in 7 samples from 7 different patients and inconsistent results were detected in 6 of these patients. A detailed description of these results is shown in Table 5.

**Table 5:** Discordant results between Aptima^®^ and Allplex^™^ assays regarding the detection of *C*. *trachomatis*, *M*. *genitalium and N*. *gonorrhoeae*.

Sample (n)	Aptima^®^	Allplex^™^
Anal (1)	CT GC	GC
Anal (2)	MG	Negative
Anal (1)	MG CT	CT
Endocervical (1)	GC	Negative
Endocervical (1)	MG	Negative
Endocervical (1)	MG CT	MG
Pharyngeal (3)	CT	Negative
Pharyngeal (2)	GC	Negative
Pharyngeal (6)	MG	Negative
Pharyngeal (2)	MG CT	CT
Urine (1)	CT	Negative
Urine (5)	GC	Negative
Urine (1)	MG	Negative
Urine (1)	MG GC	GC

**CT**, *C*. *trachomatis*; **MG**, *M*. *genitalium*; **NG**, *N*. *gonorrhoeae*.

## Discussion

The relatively high prevalence of STIs and the need for a rapid and accurate diagnostic tool for their detection justifies that any new methodology must be thoroughly evaluated before its implementation in routine laboratory practice. In this study, we evaluated the performance of TMA-based assays (Aptima Combo 2^®^ (for *C*. *trachomatis* and *N*. *gonorrhoea*), Aptima^®^ *T*. *vaginalis* and Aptima^®^ *M*. *genitalium*) on the Panther^®^ platform for simultaneous detection of *C*. *trachomatis*, *N*. *gonorrhoeae*, *T*. *vaginalis* and *M*. *genitalium* in comparison with a multiplex RT-PCR assay (Allplex^™^ STI Essential, Seegene^®^). Although Aptima^®^ assays have previously shown good performances in the diagnosis of these pathogens [10–15], this is, to our knowledge, the first time that the detection of these four pathogens together has been compared to Allplex^™^ STI Essential RT-PCR-based assay. The effectiveness of the Allplex^™^ assay has also been previously proven [20–22].

Both systems showed the highest prevalence of *C*. *trachomatis* in the analyzed samples, which is in line with this pathogen being the most frequently reported STI in Europe [23]. The sensitivity of the TMA assays was higher for most pathogens compared to multiplex RT-PCR. It is very remarkable that most of the samples responsible for this higher sensitivity were pharyngeal swab samples, and that *M*. *genitalium* was more frequently detected in this location with TMA assays. For most bacterial STI, the throat is the anatomical location with the lowest number of positive samples [24] however our findings at least in the case of *M*. *genitalium*, confirm the results of recent reports in the literature [25]. Our results confirm that other sites should always be considered based on patient’s sexual habits [26]. In fact, using urogenital samples alone overlooks many infections, resulting in many patients failing to receive proper treatment [27].

The sensitivity and specificity of these TMA assays have been evaluated previously, with sensitivities and specificities > 90% [13, 14, 28], however recently, false-negative *Chlamydia trachomatis* have been reported using Aptima Combo2^®^ assays in Finland and Sweden due to a 23S rRNA C1515T mutation [29, 30]. It is important to mention that a single genetic target region should not be trusted in molecular diagnosis of infections to avoid underdiagnosis of possible mutants, this could be a limitation of this assay, that only amplify chlamydial 23S rRNA.

Our study has two important limitations. First, we could not resolve all discordant results with a third test because our sample volume was low and we did not want to dilute the sample to prevent losing sensitivity. Thereof, we could not calculate the positive predictive value and negative predictive value of this test, and we could not rule out that the higher sensitivity of the TMA assays could be due to false positive detections. Second, *T*. *vaginalis* specimens found in pharyngeal swabs with the RT-PCR test were not included in the final analysis. In fact, we found 19 pharyngeal samples that were scored positive for *T*. *vaginalis* only with the Allplex^™^ test; it is well documented that DNA-based methods may lack specificity for discriminating *T*. *vaginalis* from some other oral *Trichomonas* species existing in the pharyngeal microbiota, such as *Trichomonas tenax* [31, 32].

Our results confirm the effectiveness of the Aptima^®^ assays for STI diagnosis, providing additional evidence supporting the implementation of this methodology for routine testing in clinical diagnostics. Importantly, this TMA technology provides important features for laboratory automation and daily clinical practice. Thus, patients might benefit highly from this technology, as they can be evaluated and diagnosed at the same medical visit.

## Supporting information

S1 Table Details regarding the number of positive and negative samples identified with Aptima^®^ and Allplex^™^ assays. (DOCX)
