# Supplementary material for: Comparison between Aptima Assays (Hologic) and the Allplex STI Essential Assay (Seegene) for the diagnosis of Sexually transmitted infections
Source: PLoS One. 2019 Sep 12;14(9):e0222439. doi: 10.1371/journal.pone.0222439 (PMC6742210; doi:10.1371/journal.pone.0222439)
Supplement: S1 Table — (DOCX) [file pone.0222439.s001.docx]

**Supplementary Table 1**. Details regarding the number of positive and negative samples identified with Aptima® and Allplex^TM^ assays.

|  | | **Aptima**® | | |
| --- | --- | --- | --- | --- |
| ***C. trachomatis*** | | **+** | **-** | **Total** |
| **Allplex^TM^** | **+** | 31 | 0 | 31 |
|  | **-** | 6 | 585 | 591 |
|  | **Total** | **37** | **585** | **622** |
| ***N. gonorrhoeae*** | |  | |  |
| **Allplex^TM^** | **+** | 21 | 0 | 21 |
|  | **-** | 8 | 593 | 601 |
|  | **Total** | **29** | **593** | **622** |
| ***M. genitalium*** | |  | |  |
| **Allplex^TM^** | **+** | 10 | 0 | 10 |
|  | **-** | 14 | 598 | 612 |
|  | **Total** | **24** | **598** | **622** |
| ***T. vaginalis*** | |  | |  |
| **Allplex^TM^** | **+** | 5 | 0 | 5 |
|  | **-** | 0 | 617 | 617 |
|  | **Total** | **5** | **617** | **622** |
